# Quantifying the Predictive Accuracy of a Polygenic Risk Score for Predicting Incident Cancer Cases : Application to the CARTaGENE Cohort

**DOI:** 10.3389/fgene.2020.00408

**Published:** 2020-04-24

**Authors:** Julianne Duhazé, Rodolphe Jantzen, Yves Payette, Thibault De Malliard, Catherine Labbé, Nolwenn Noisel, Philippe Broët

**Affiliations:** ^1^Research Center, CHU Sainte-Justine, Montreal, QC, Canada; ^2^University Paris-Saclay, CESP, INSERM, Villejuif, France; ^3^CARTaGENE, CHU Sainte-Justine, Montreal, QC, Canada

**Keywords:** pseudo-*R*^2^, polygenic risk score, survival models, survival mixture model, breast cancer

## Abstract

With the increasing use of polygenic risk scores (PRS) there is a need for adapted methods to evaluate the predictivity of these tools. In this work, we propose a new pseudo-*R*^2^ criterion to evaluate PRS predictive accuracy for time-to-event data. This new criterion is related to the score statistic derived under a two-component mixture model. It evaluates the effect of the PRS on both the propensity to experience the event and on the dynamic of the event among the susceptible subjects. Simulation results show that our index has good properties. We compared our index to other implemented pseudo-*R*^2^ for survival data. Along with our index, two other indices have comparable good behavior when the PRS has a non-null propensity effect, and our index is the only one to detect when the PRS has only a dynamic effect. We evaluated the 5-year predictivity of an 18-single-nucleotide-polymorphism PRS for incident breast cancer cases on the CARTaGENE cohort using several pseudo-*R*^2^ indices. We report that our index, which summarizes both a propensity and a dynamic effect, had the highest predictive accuracy. In conclusion, our proposed pseudo-*R*^2^ is easy to implement and well suited to evaluate PRS for predicting incident events in cohort studies.

## 1. Introduction

With the power of genotyping technologies, genome-wide association studies (GWAS) focusing on complex diseases have identified a large number of genetic variants associated with various traits of interest (e.g., diabetes, cardiovascular diseases, cancer…) (Buniello et al., 2019). These large GWAS have provided various lists of disease-related single nucleotide polymorphisms (SNP) together with their effect size estimates (McCarthy et al., 2008). Based on these findings, there has been a growing interest recently in deriving polygenic risk scores (PRS) in order to provide individual risk predictions for various phenotypes (Torkamani et al., 2018). Broadly speaking, for each individual, a classical polygenic score consists of a linear combination of the trait-associated alleles carried by the subject and weighted by their effect sizes. The list of these risk alleles and their corresponding weights are obtained from published GWAS. Keeping in mind classical modeling assumptions of linearity, additivity, and lack of interactions, these PRS provide some estimates of the probabilistic susceptibility of an individual to experience the disease and can be used for disease risk stratification (Torkamani et al., 2018). As a result, in recent years, a burgeoning literature has started to focus on the evaluation of published PRS (for a few: International Multiple Sclerosis Genetics Consortium et al., 2010; Machiela et al., 2011; Khera et al., 2018).

The predictive accuracy of these PRS are usually assessed by measures such as the coefficient of determination (noted as *R*^2^) which is interpreted as the proportion of variation in the phenotype that is explained by the PRS. For continuous outcomes, the coefficient of determination is well-defined and unique, however its extension to other outcomes such as binary outcomes is not straightforward. By analogy with the linear model and from different perspectives, various generalizations of the *R*^2^ have been proposed (e.g., Hu et al., 2006). They are usually referred to as pseudo-*R*^2^.

In practice, most of the pseudo-*R*^2^ that are used for assessing the predictive accuracy of PRS focus on binary outcomes. This is the case for cancer susceptibility where most of the PRS studies have analyzed cancers as a binary outcome (affected/not affected), irrespective of the age of onset. However, as the occurrence of cancers is strongly influenced by age, prediction modeling should not neglect the dynamic of cancer occurrence over time. Thus, the assessment of predictive accuracy of PRS for cancer susceptibility based upon epidemiological cohorts should use time-to-event data and provide predictivity for a defined risk projection interval.

For such quantification with time-to-event data, a large spectrum of pseudo-*R*^2^ have been proposed, most of them relying on the classical Cox proportional hazards model (Cox, 1972). These pseudo-*R*^2^ fall mainly into the categories of explained randomness (entropy-based) or explained variation (variance-based). This latter framework corresponds to the proportion of the outcome variance that is explained by the studied covariates. The estimates rely either on comparing empirical survival functions with and without covariates or on statistical quantities which are directly or indirectly related to the likelihood function (O'Quigley, 2008; Flandre et al., 2017). In practice, most of these pseudo-*R*^2^ have to maximize the log (partial) likelihood of the full model. However, it is sometimes not a straightforward issue, particularly for complex non-proportional survival models.

We had to face such issue in a recent study relying upon the Quebec population-based cohort CARTaGENE where our main objective was to evaluate the 5-year predictivity of a published PRS for breast cancer. To assess the 5-year predictivity, we had to rely upon a non-standard survival model that considers age as the time scale and takes into account that a proportion of the individuals are not susceptible to develop the disease within 5 years. Such survival model belongs to the class of survival mixture models where the population under study is a mixture of individuals with those at risk for experiencing the event within 5 years and those who are not at risk (Maller and Zhou, 1996). The motivation behind the use of this survival model is that at a specified age and over a 5-year horizon a woman can be either susceptible or non-susceptible to experience breast cancer and her PRS may be related to the propensity for experiencing the event. Moreover, among the susceptible ones, some of them may experience the event earlier than others and their PRS may be linked to the dynamic of the event. In this context, we know that the classical survival models are not well suited for quantifying both effects.

This issue prompted us to derive a pseudo-*R*^2^ which relies on time-to-event data and quantifies the 5-year predictivity accuracy of PRS. This new criterion extends a previous work (Rouam et al., 2010) on pseudo-*R*^2^ that was restricted to classical proper survival models. The score statistic derived from the partial likelihood under an entry-age-stratified age-scaled working survival model enables the calculation of the pseudo-*R*^2^. This criterion quantifies the predictive ability of the studied factor to separate subject outcomes on both the probability of experiencing the event and the occurrence dynamic.

In this work, we present this new criterion and report the results obtained from a simulation study. We show its practical interest for evaluating the accuracy of published PRS for predicting incident breast cancer cases in the following 5 years using the Quebec population-based cohort CARTaGENE.

## 2. Materials and Methods

### 2.1. Materials: The CARTaGENE Study

CARTaGENE (www.cartagene.qc.ca) is a population cohort consisting of 43,037 Quebec residents aged between 40 and 69 years at recruitment (Awadalla et al., 2013). Enrollment of participants began in July 2009 and was carried out in two phases in six metropolitan areas. On their enrollment date, each participant filled out a questionnaire about health, lifestyle, individual, and familial history of disease, and prescribed medication. Only the women were included in the breast cancer study (*n* = 23,797).

Linkage with administrative databases was included in the participant consent form: 1/the Quebec administrative health database MED-ÉCHO contains hospitalizations, claims, and date of death of insured patients (about 98% of Quebec residents) (data available from January 1st, 1998 to March 31st, 2016); 2/the Quebec Breast Cancer Registry contains information about the Quebec Breast Cancer Screening Program, such as mammogram results and breast cancers histological confirmation (data available from May 15th, 1998 to December 31st, 2017).

To comprehensively define women with a breast cancer (invasive or *in situ*), we used an algorithm based on a previous report from the Institut National de Santé Publique du Québec (INSPQ) (Théberge et al., 2003). Using the Breast Cancer Registry, we retrieved the incidence date of histologically confirmed breast cancers. Then, we selected all women having an abnormal mammography and retrieved, if available, the incidence date from the MED-ÉCHO database for women with at least two claims in 2 years or one hospitalization with the corresponding ICD-9 (174, 2330) and ICD-10 (C50, D05) codes.

For the breast cancer study, only the women with genetic data available and who did not have a breast cancer before their enrollment in the CARTaGENE cohort were selected (*n* = 4,554).

### 2.2. Polygenic Risk Scores

Based on the study of Evans et al. (2017), we computed a PRS for each woman. This PRS uses 18 SNP that have been shown to be associated with breast cancer risk in general European populations together with the published per-allele ORs. Each PRS is the linear combination of the number of risk alleles for each of the 18 SNP weighted by their corresponding log odds-ratios.

Genotyping data has been generated through different projects and using different chips and platforms (Illumina Omni 2.5M, Illumina Infinium Global Screening Array, and Affymetrix Axiom UK biobank). Among the 18 SNP composing the Evans PRS, three were not available in our study and nine had missing data. To impute the missing SNP, we used the Michigan Imputation Server with the Minimac4 algorithm (Das et al., 2016). Imputation reference panel was the HRC r1.1 2016 European population, and the phasing was performed with Eagle v2.4 (Loh et al., 2016). A genetic quality control (QC) was made before the imputation. After imputation, QC was performed on individuals based on the Anderson et al. protocol (Anderson et al., 2010), using all individuals genotyped with the Illumina Infinium Global Screening Array. Individuals with a call rate lower than 95% and a heterozygosity higher than three standard deviations were removed. In pairs of individuals with an identity by state (IBD) higher than 0.1875, the individual with the lowest call rate was removed. To remove participants with divergent European ancestries, we used the first two principal components with the HapMap phase 3 reference panel (The International HapMap 3 Consortium, 2010).

All the QC procedures were performed using PLINK (Purcell et al., 2007) and R package Gaston (Perdry and Dandine-Roulland, 2019).

### 2.3. Outcomes

The outcome was the age at occurrence of breast cancer. Patients without breast cancer occurrence were censored. Censoring time was age at the end of the 5-year study period (administrative censoring) or age at death.

For taking into account the age effect, our survival model was stratified by age at cohort entry with four groups: [39–50], [50–55], [55–60], [60–69].

### 2.4. Methods: The Pseudo-R^2^ Criterion

#### 2.4.1. Notations and Survival Model

In this work, we considered a survival model with age as the time scale and stratification on age at entry in the study. Let *A*_0_ denote a random variable that corresponds to the age at which the individual free of disease enters the cohort. Let *A* be the age at which the individual is experiencing the event of interest with $A=A_{0}+T^{⋆}$ where *T*^⋆^ is the event time (i.e., the time elapsed between the enrollment in the cohort and the date of event). Let *C* be the age at which the individual is censored with $C=A_{0}+C^{⋆}$ where *C*^⋆^ is the censoring time (i.e., the time elapsed between the enrollment in the cohort and the date of analysis or last follow-up). Conditionally on *A*_0_, let the random variables *A* and *C* assumed to satisfy the condition of independent censoring (Fleming and Harrington, 2005).

In the following, we consider *J* strata for age at entry. For a subject *i* in stratum *j* (*j* = 1, .., *J*), let *X*_*ij*_ = min(*A*_*ij*_, *C*_*ij*_) be the observed follow-up time on the age scale, δ_*ij*_ = **1**_(_*X*__*ij*_ = *A*_*ij*_)_ the indicator of event and *Y*_*ij*_(*s*) = **1**_(_*X*__*ij*_ ≥ *s*)_ the indicator of being at risk for the event at age *s*. *Y*_*ij*_(*s*) = 1 indicates that subject *i* in stratum *j* is at risk just before time *s*, *Y*_*ij*_(*s*) = 0 otherwise. Let *Z*_*ij*_ be the value of the PRS computed for individual *i* in stratum *j*. For each individual, the observed data consists of (*X*_*ij*_, δ_*ij*_,_*A*_0_*ij*_, *Z*_*ij*_).

Since we focus on a 5-year projection interval, we have to take into account that some individuals who enter the cohort are non-susceptible subjects for the event of interest whereas the other are susceptible subjects who may experience the event by the end of the 5 years or may be censored prior to experiencing the event.

Thus, we consider the following two-part semi-parametric multiplicative model whose marginal survival distribution is expressed as:

(1) $$\begin{array}{l} S\left(a|j,Z=z\right)=\exp\left[-\theta_{j}e^{\alpha z}\right]+\left\{1-\exp\left[-\theta_{j}e^{\alpha z}\right]\right\}\\ \;\times \exp\left[-\Lambda_{0j}\left(a\right)e^{\beta z}\right] \end{array}$$

where $\exp\left[-\theta_{j}e^{\alpha z}\right]$ is the probability of being a non-susceptible individual for a subject in stratum *j*. This latter quantity (sometimes called the tail defect) depends upon the age at entry and the PRS. Here, θ_*j*_ > 0 is an unknown positive age-stratum parameter and α an unknown parameter of interest which quantifies the impact of the PRS on the propensity of being a non-susceptible individual.

In this model, $\exp\left[-\Lambda_{0j}\left(a\right)e^{\beta z}\right]$ is the conditional survival distribution of time-to-event for stratum *j* for those who are susceptible to experience the event within the projection interval. Here, Λ_0*j*_(*a*) is an unspecified conditional baseline cumulative hazard function for stratum *j* and β is an unknown parameter of interest which quantifies the impact of the PRS on the dynamic of the event's occurrence.

In the following, we re-write the survival model presented above in terms of the multiplicative hazard functions as:

$$\begin{array}{l} h\left(a|j,Z=z\right)=\lambda_{0j}\left(a\right)\left(1-e^{-\theta_{j}e^{\alpha z}}\right)e^{\beta z}e^{-\Lambda_{0j}\left(a\right)e^{\beta z}}e^{-log\left(S\left(a|j,z\right)\right)} \end{array}$$

where λ_0*j*_(*a*) is the first derivative of Λ_0*j*_(*a*).

From this multiplicative model, a partial likelihood can be written as follows:

$$\begin{array}{l} L(\alpha ,\beta ,\theta_{j},\Lambda_{0j})=\prod_{j=1}^{J}\{\prod_{i=1}^{n_{j}}{\left[\frac{(1-e^{-\theta_{j}e^{\alpha z_{i}}})e^{\beta z_{i}}e^{-\Lambda_{0}{\;}_{j}(a_{i})e^{\beta z_{i}}}e^{-log(S_{j}(a_{i}|z_{i}))}}{\sum_{m=1}^{n_{j}}Y_{m}(a_{i})(1-e^{-\theta_{j}e^{\alpha z_{m}}})e^{\beta z_{m}}e^{-\Lambda_{0}{\;}_{j}(a_{i})e^{\beta z_{m}}}e^{-log(S_{j}(a_{i}|z_{m}))}}\right]}^{\delta_{i}}\} \end{array}$$

with *n*_*j*_ the number of individuals in stratum *j* and *N* = *n*_1_+…+*n*_*J*_ the total number of individuals. Here, *S*_*j*_(*a*) and Λ_0*j*_(*a*) are the marginal survival function and the conditional baseline cumulative hazard function for stratum *j*, respectively.

#### 2.4.2. Scores Components

From what precedes, we can easily obtain the two components of the log partial likelihood score function evaluated under the null hypothesis of no PRS effect $H_{0}:\beta =\alpha =0$:

$$\begin{array}{l} U_{1ij}=\frac{\partial LL_{ij}}{\partial \alpha }=\delta_{i}w_{1}(a_{i})\left\{z_{i}-\frac{\sum_{l\in {ℛ}_{ij}}Y_{l}(a_{i})w_{1}(a_{i})z_{l}}{\sum_{l\in {ℛ}_{ij}}Y_{l}(a_{i})w_{1}(a_{i})}\right\}\\ U_{2ij}=\frac{\partial LL_{ij}}{\partial \beta }=\delta_{i}w_{2}(a_{i})\left\{z_{i}-\frac{\sum_{l\in {ℛ}_{ij}}Y_{l}(a_{i})w_{2}(a_{i})z_{l}}{\sum_{l\in {ℛ}_{ij}}Y_{l}(a_{i})w_{2}(a_{i})}\right\} \end{array}$$

where $R_{ij}$ is the risk set of stratum *j* including the individual that experienced the event at time *a*_*i*_. Here $w_{1}\left(s\right)=e^{-\theta_{j}}\theta_{j}S_{j}^{-1}\left(s\right)$ and $w_{2}\left(s\right)=\left[e^{-\theta_{j}}\left(1-e^{-\Lambda_{0j}\left(s\right)}-\Lambda_{0j}\left(s\right)\right)+e^{-\Lambda_{0j}\left(s\right)}\right]S_{j}^{-1}\left(s\right)$ are the weights for the two components, respectively. The nuisance parameters, $e^{-\theta_{j}}$, Λ_0*j*_ and *S*_*j*_(*s*) are the tail defect, the conditional cumulative hazard function and the marginal survival distribution for stratum *j* computed under the null hypothesis.

From a biological perspective, the quantity *U*_1*ij*_ can be linked to differences in the propensity of being a non-susceptible individual and *U*_2*ij*_ to differences in the dynamic of the occurrence of the event of interest for susceptible individuals.

#### 2.4.3. Scores as Measures of Separability

Following a previous work (Rouam et al., 2010), the non-null quantities *U*_1*ij*_ and *U*_2*ij*_ computed at event time can be reformulated as two measures of separability that quantify the ability of the PRS to separate individuals from the stratum *j* who experience the event at time *a*_*i*_ from those who are still at risk.

The first quantity *U*_1*ij*_ can be re-written as:

$$\begin{array}{l} U_{1ij}=\delta_{i}w_{1}(a_{i})\left(\frac{\sum_{l\in {ℛ}_{ij}^{*}}Y_{l}(a_{i})w_{1}(a_{i})}{\sum_{l\in {ℛ}_{ij}}Y_{l}(a_{i})w_{1}(a_{i})}\right)\\ \;\times \;\left\{z_{i}-\frac{\sum_{l\in {ℛ}_{ij}^{*}}Y_{l}(a_{i})w_{1}(a_{i})z_{l}}{\sum_{l\in {ℛ}_{ij}^{*}}Y_{l}(a_{i})w_{1}(a_{i})}\right\}\\ U_{1ij}=\delta_{i}w_{1}(a_{i})\left(\frac{\sum_{l\in {ℛ}_{ij}^{*}}Y_{l}(a_{i})w_{1}(a_{i})}{\sum_{l\in {ℛ}_{ij}}Y_{l}(a_{i})w_{1}(a_{i})}\right)\times \left(z_{i}-{\bar{z}}_{w_{1}}\right) \end{array}$$

In like manner, the second one is as:

$$\begin{array}{l} U_{2ij}=\delta_{i}w_{2}(a_{i})\left(\frac{\sum_{l\in {ℛ}_{ij}^{*}}Y_{l}(a_{i})w_{2}(a_{i})}{\sum_{l\in {ℛ}_{ij}}Y_{l}(a_{i})w_{2}(a_{i})}\right)\times \left(z_{i}-{\bar{z}}_{w_{2}}\right) \end{array}$$

where $R_{i}^{*}$ is the risk set of stratum *j* without the individual that experienced the event at time *a*_*i*_.

The two non-null components *U*_1*ij*_ and *U*_2*ij*_ can be interpreted as a weighted differences between the mean value of the genomic score for the individuals who experience the event at time *a*_*i*_ and the weighted mean for those who are still at risk (i.e., the mixture of individuals who do not experience the event at time *a*_*i*_). Differences close to zero indicate a weak or null separability. Large differences indicate that the individuals are well-separated.

#### 2.4.4. Shifted Score Components

In the following, we introduce the shifted scores (or robust scores) *W*_1*ij*_ and *W*_2*ij*_ derived from the seminal work of Lin and Wei (1989). The shifts take into account the dependence between the individuals scores *U*_1*ij*_ and *U*_2*ij*_. The shifted scores *W*_1*ij*_ and *W*_2*ij*_ are independent and identically distributed (Lachin, 2011) and are as:

$$\begin{array}{l} W_{1ij}=U_{1ij}-{\tilde{U}}_{1ij}=\\ \;\delta_{i}w_{1}(a_{i})\left(z_{i}-\frac{\sum_{l\in {ℛ}_{ij}}Y_{l}(a_{i})w_{1}(a_{i})z_{l}}{\sum_{l\in {ℛ}_{ij}}Y_{l}(a_{i})}\right)\\ \;-\sum_{l=1}^{n_{j}}\frac{Y_{i}(t_{l})\delta_{l}w_{1}(a_{l})}{\sum_{l\in {ℛ}_{ij}}Y_{r}(a_{l})}\left(z_{i}-\frac{\sum_{l\in {ℛ}_{ij}}Y_{r}(a_{l})w_{1}(a_{l})z_{r}}{\sum_{l\in {ℛ}_{ij}}Y_{r}(a_{l})}\right)\\ W_{2ij}=U_{2ij}-{\tilde{U}}_{2ij}=\\ \;\delta_{i}w_{2}(a_{i})\left(z_{i}-\frac{\sum_{l\in {ℛ}_{ij}}Y_{l}(a_{i})w_{2}(a_{i})z_{l}}{\sum_{l\in {ℛ}_{ij}}Y_{l}(a_{i})}\right)\\ \;-\sum_{l=1}^{n_{j}}\frac{Y_{i}(t_{l})\delta_{l}w_{2}(a_{l})}{\sum_{l\in {ℛ}_{ij}}Y_{r}(a_{l})}\left(z_{i}-\frac{\sum_{l\in {ℛ}_{ij}}Y_{l}(a_{i})w_{2}(a_{l})z_{r}}{\sum_{l\in {ℛ}_{ij}}Y_{r}(a_{l})}\right) \end{array}$$

The practical expressions of these latter quantities are obtained by plugging the two estimates ŵ_1_(*s*) and ŵ_2_(*s*) where we replace *S*_*j*_(*s*) by the left-continuous version of the Fleming-Harrington estimator obtained under $H_{0}$ using the Nelson-Aalen estimator. The nuisance parameter θ is estimated by $\hat{\theta_{j}}=-log\left(1-S_{j}\left(t_{\max}\right)\right)$ where *t*_max_ is the last observed failure time. The shifts ${Ũ}_{1ij}=\hat{\text{E}}\left({Û}_{1ij}\right)$ and ${Ũ}_{2ij}=\hat{\text{E}}\left({Û}_{1ij}\right)$ are weighted average of the score calculated at times *s* prior to time *a*_*i*_.

In the following, as we focus on separability measures we will consider only the shifted score components associated with event times that we denoted as $W_{1ij}^{*}$ and $W_{2ij}^{*}$.

#### 2.4.5. Pseudo-*R*^2^ Criterion

In the following we derive a pseudo-*R*^2^ criteria which is interpreted as the proportion of variation of separability that is explained by the PRS. Since the classical score contributions (*U*_*ij*_) are based on a partial rather than a full likelihood, they are not independently and identically distributed. However, the shifted score contributions (*W*_*ij*_) introduced by Lin and Wei (1989) are independent and identically distributed. As our pseudo-*R*^2^ quantifies the proportion of variance explained by the PRS, it relies on variance estimates and this latter condition is important for estimation purposes. Moreover, since our individual score contributions can be expressed as differences between the means of the PRS of the group of patients observed experiencing the event of interest and the group of those observed not experiencing the event, the considered shifted score contributions are those calculated at each occurrence of the event.

We recall that the quantities $W_{1ij}^{*}$ and $W_{2ij}^{*}$ represent the measures of separability that are calculated at an event time. We denote *k*_*j*_ the number of event times for stratum *j*. The total number of events is denoted by *K*. In practice, we use the generalized variance of the two-dimensional random vector $W=\left(W_{1}^{*},W_{2}^{*}\right)$ that is defined as the determinant of its variance-covariance matrix. Then, we derive a pseudo-R^2^ which is based on the relative difference between an estimate of the variance-covariance matrix of the shifted scores computed under the null hypothesis ($H_{0}$: no effect of the PRS; *E*[*W*] = 0) and an estimate calculated under the alternative hypothesis ($H_{1}$: effect of the PRS; *E*[*W*] ≠ 0).

Under the null hypothesis ($H_{0}:\alpha =\beta =0$), we have:

$$\begin{array}{l} {\hat{\sigma }}^{2}(W_{1}^{*})=\frac{1}{K-1}\sum_{j=1}^{J}\left(\sum_{i=1}^{k_{j}}W_{1ij}^{*}{\;}^{2}\right)\\ \hat{\sigma }(W_{12}^{*})=\frac{1}{K-1}\sum_{j=1}^{J}\left(\sum_{i=1}^{k_{j}}W_{1ij}^{*}W_{2ij}^{*}\right)\\ {\hat{\sigma }}^{2}(W_{2}^{*})=\frac{1}{K-1}\sum_{j=1}^{J}\left(\sum_{i=1}^{k_{j}}W_{2ij}^{*}{\;}^{2}\right). \end{array}$$

Then (omitting the term $\frac{1}{K-1}$) we have :

$$\begin{array}{l} \det(\Sigma )=\sum_{j=1}^{J}\left(\sum_{i=1}^{k_{j}}W_{1ij}^{*}{\;}^{2}\right)\sum_{j=1}^{J}\left(\sum_{i=1}^{k_{j}}W_{2ij}^{*}{\;}^{2}\right)\\ \;-(\sum_{j=1}^{J}\left(\sum_{i=1}^{k_{j}}W_{1ij}^{*}W_{2ij}^{*}\right){)}^{2} \end{array}$$

Under the alternative hypothesis ($H_{1}$), we have:

$$\begin{array}{l} {\hat{\sigma }}^{2}(W_{1}^{*})=\frac{1}{K-1}\sum_{j=1}^{J}\left(\sum_{i=1}^{k_{j}}W_{1ij}^{*}{\;}^{2}-\frac{1}{k_{j}}\sum_{i=1}^{k}{\left(W_{1ij}^{*}\right)}^{2}\right)\\ \hat{\sigma }(W_{12}^{*})=\frac{1}{K-1}\sum_{j=1}^{J}\;\\ \left(\sum_{i=1}^{k_{j}}W_{1ij}^{*}W_{2i}^{*}-\frac{1}{k_{j}}\{\sum_{i=1}^{k}W_{1ij}^{*}\sum_{i=1}^{k}W_{2ij}^{*}\}\right)\\ {\hat{\sigma }}^{2}(W_{2}^{*})=\frac{1}{K-1}\sum_{j=1}^{J}\left(\sum_{i=1}^{k_{j}}W_{2ij}^{*}{\;}^{2}-\frac{1}{k_{j}}\sum_{i=1}^{k}{\left(W_{2ij}^{*}\right)}^{2}\right). \end{array}$$

Then:

$$\begin{array}{l} \det(\Sigma^{⋆})=\sum_{j=1}^{J}\left(\sum W_{1ij}^{*}{\;}^{2}-\frac{1}{k_{j}}{\left(\sum W_{1ij}^{*}\right)}^{2}\right)\\ \;\times \sum_{j=1}^{J}\left(\sum W_{2ij}^{*}{\;}^{2}-\frac{1}{k_{j}}{\left(\sum W_{2ij}^{*}\right)}^{2}\right)\\ \;-{\left(\sum_{j=1}^{J}\left(\sum W_{1ij}^{*}W_{2ij}^{*}-\frac{1}{k_{j}}\left(\sum W_{1ij}^{*}\sum W_{2ij}^{*}\right)\right)\right)}^{2} \end{array}$$

Finally, $\Delta =\frac{\det\left(\Sigma \right)-\det\left(\Sigma^{⋆}\right)}{\det\left(\Sigma \right)}$ is the pseudo-*R*^2^ criterion.

This quantity measures the global predictive accuracy of the PRS. As shown from the Supplementary Material and the simulation study, the pseudo-*R*^2^ is unit-less, ranges from zero to one, increases with the effect related with either the tail defect proportion or the dynamic of the occurrence of the event of interest.

### 2.5. Simulation-Based Studies

The objective of this section is to evaluate the behavior of the proposed index Δ for different levels of tail defect, values of parameters α and β, sample sizes *n* and percentages of censoring. We compare the values of Δ to those of the pseudo-*R*^2^ proposed by Nagelkerke (*N*) based on a transformation of the partial likelihood ratio test (Nagelkerke, 1991), by O'Quigley and Flandre (*OF*) based on Schoenfeld residuals (O'Quigley and Flandre, 1994), by Xu and O'Quigley (*XO*) (Ronghui Xu and O' quigley, 1999), by O'Quigley et al. (*OXS*) (O'Quigley et al., 2005) based on explained randomness measures relying on a transformation of the Kullback-Leibler information gain and by Rouam et al. (*RMB*) (Rouam et al., 2010) based on the robust score statistic. These indexes are implemented in the R packages “survAUC” (Potapov et al., 2015) (for *N*, *XO*, and *OXS*), “PHeval” (Chauvel, 2018) (for *OF*), and “survival” (Therneau, 2015) (for *RMB*).

For the sake of simplicity and without loss of generality, we evaluated the behavior of the test with only one stratum for most of the simulation scenarios. However, in order to check the behavior of the indice with strata, we performed an additional simulation scheme in a case with two strata.

#### 2.5.1. Simulation Scheme

Survival times were generated according to the two-part survival model presented in the previous section with baseline conditional cumulative hazard function Λ_0_(*s*) = *s*. The baseline probability of being a non-susceptible was chosen such as *exp*(−θ) was equal to 0.3, 0.5, or 0.7. For each subject, we simulated a variable *Z* (its PRS) from a standard Normal distribution [N(0,1)].

In order to see the behaviors of the different indices and in particular if they were able to attain values close to one, the following configurations were considered for the hazard ratio (HR) values *e*^α^ and *e*^β^ (termed as “*propensity effect”* and “*dynamic effect”*, respectively): 1 (no effect), 1.5, 2, 2.5, 3, 4, 5, 10, 20, 50, or 500. These values explore a large range of effects from small [*log*(1.5) ≈ 0.41] to huge [*log*(500) ≈ 6.21] for α and β. The number of subjects was 500.

For investigating the robustness of the proposed pseudo-*R*^2^ to model misspecification, we performed simulations with survival times generated according to an improper Gompertz distribution such as *S*(*s*|*Z* = *z*) = *exp*(−θ*e*^α*z*^(1−*exp*(−*se*^β*z*^)).

For investigating the robustness of the indice to the distribution of the covariate, we simulated a variable *Z* from a Student distribution with ten degrees of freedom. The effect of censoring was investigated by generating independently censoring times from a uniform distribution over [0, *u*]. Values for *u* were computed from the chosen percentage of censoring and from the parameters of the considered distributions. The percentage of censoring refers to the percentage of censored observations without the fraction of non-susceptible subjects. Here, 20% censoring were considered.

For all these simulations, values *e*^α^ and *e*^β^ were :1, 1.25, 1.5 and 1, 1.5, 2, respectively. The number of subjects was 500.

For investigating the robustness of the proposed pseudo-*R*^2^ to the number of subjects, we performed additional simulations with 200, 500, and 1, 000 subjects. We also performed additional simulations where we generated a stratification variable from a Bernoulli distribution with parameter 0.5.

For each configuration, 1, 000 replications were performed.

## 3. Results

### 3.1. Simulation Results

**Figure 1:**
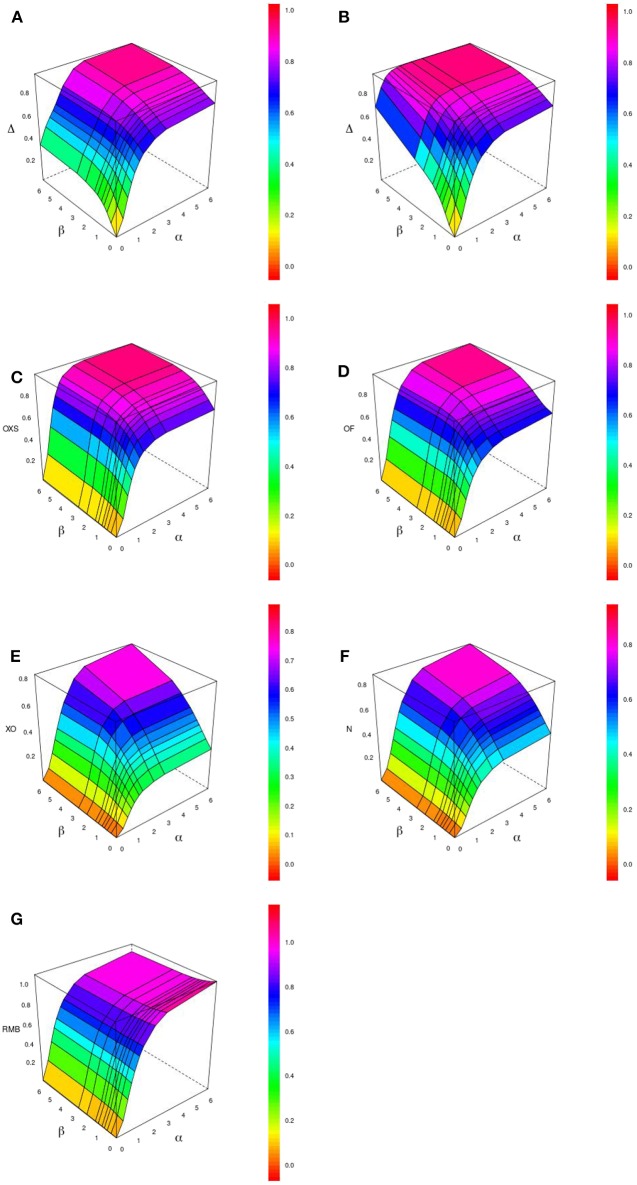
Display of the behavior of different pseudo-*R*^2^ indices when the effects of the evaluated criterion on the propensity (α) and on the dynamic (β) vary. The graphic shows Δ with 0% **(A)** and 20% **(B)** censoring and other indexes with 0% censoring : *OXS* **(C)**, *OF* **(D)**, *XO* **(E)**, *N* **(F)**, and *RMB* **(G)**, for 500 subjects and a tail defect of 70%.

**Figure 2:**
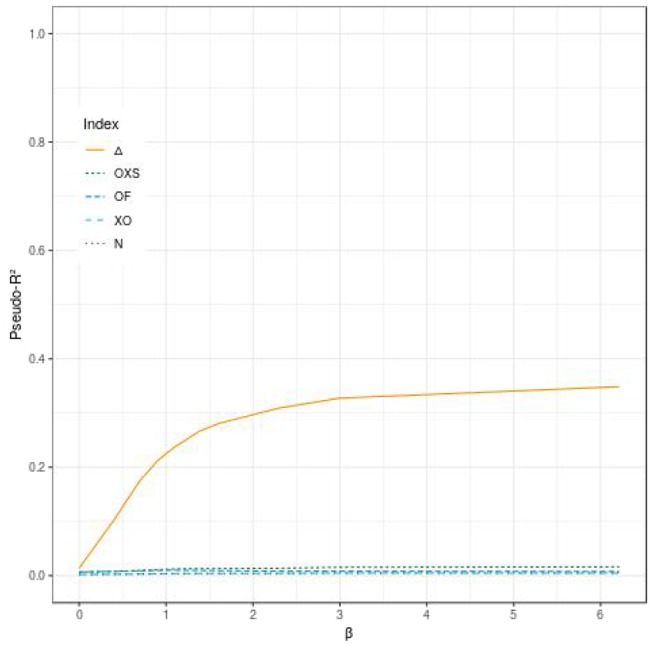
Comparison of the evolution of different pseudo-*R*^2^ indices according to the effect of the evaluated polygenic risk score on the dynamic (β) when the criterion has no effect on the propensity (α), for 500 subjects, a tail defect of 70% and no censoring.

Figure 1 displays the behavior of Δ, *OF*, *OXS*, *XO*, *N*, and *RMB* according to α and β for a tail defect of 70% with 500 subjects and no censoring. Our simulations showed that the value of Δ increases with the value of the strength of the PRS' effect (through the hazards ratio *e*^α^ and *e*^β^). In our simulation study, Δ ranges from 0 when both α and β are null, to near 1 for very large effects of α and β. In the range of effects presented in this paper, the maximum value reached by Δ is 0.96 when both α and β take value of 6.21 (corresponding to a HR of 500) (see Figure 1A). We can see that Δ is able to quantify an effect on the dynamic (β) when there is no propensity effect (α) related to the PRS. For example, when α is null and β = 0.92 (corresponding to a HR of *e*^β^ = 2.5) we have Δ = 0.52. The other studied indices are not able to quantify a dynamic effect when there is no propensity effect (see Figures 1A,C–F, 2).

**Figure 3:**
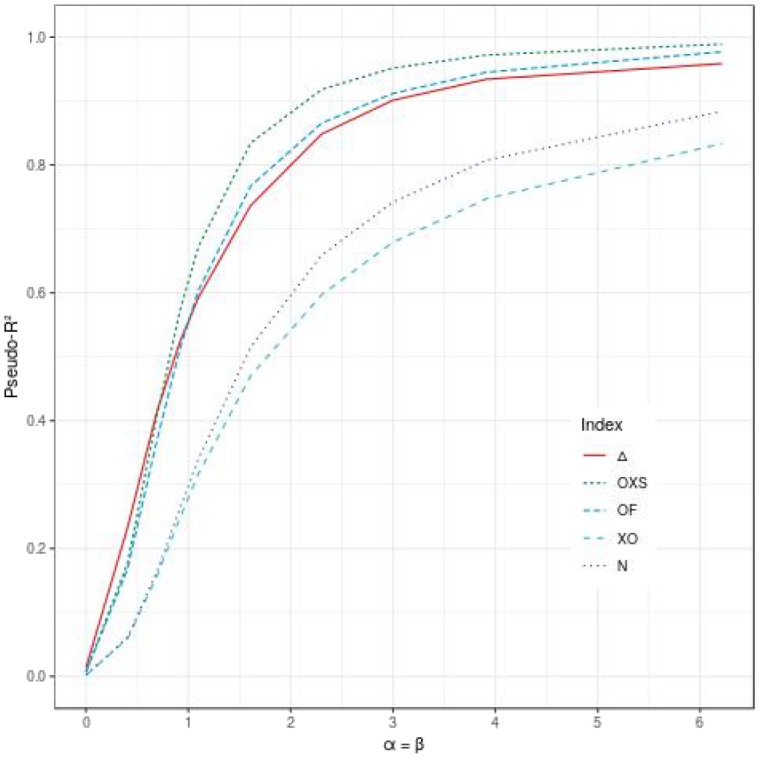
Comparison of the evolution of different pseudo-*R*^2^ indices according to the effect of the evaluated polygenic risk score on the dynamic (β) and the propensity (α), when α = β, for 500 subjects, a tail defect of 70% and no censoring.

When the propensity effect is not null, all indices, including ours, are able to quantify the mixture of both effects. The different configurations show that Δ, *OF*, and *OXS* lead to higher values than *XO* and *N*. Figure 3 displays the values of the indices according to the propensity and dynamic effects, when both the parameters α and β have the same value. Δ has the highest values when α = β ≤ 0.91 (*e*^α^ = *e*^β^ ≤ 2.5) and ranks in third place for higher values of α and β.

**Figure 4:**
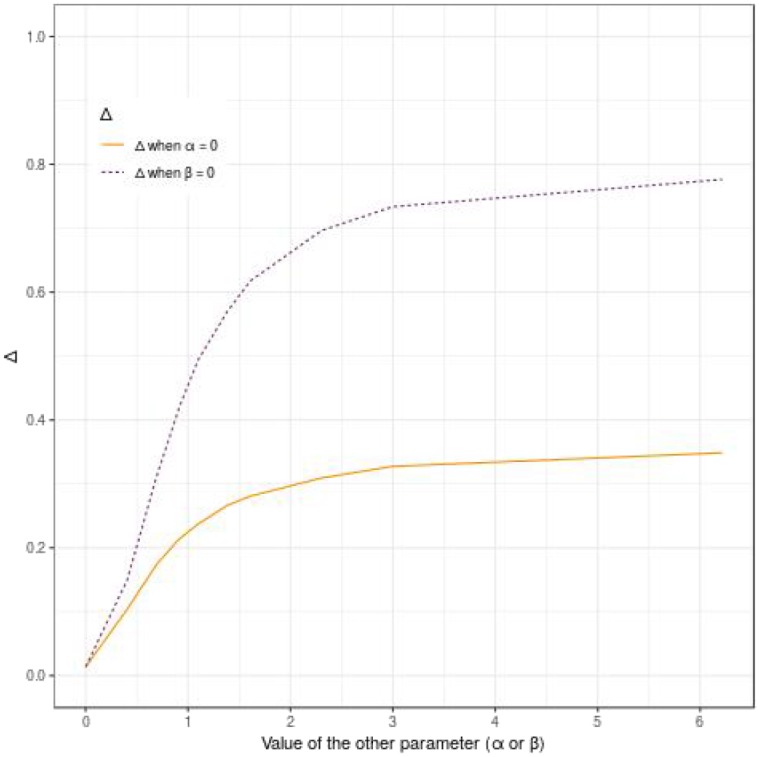
Display of the behavior of the pseudo-*R*^2^ Δ according to the effect of the evaluated polygenic risk score on the dynamic (β) or the propensity (α), when α or β is null, for 500 subjects, a tail defect of 70% and no censoring.

Figure 4 shows that Δ quantifies both the propensity effect and the dynamic effect. However, the reported predictive accuracy is not symmetrical and is higher for the propensity effect (α) as compared to the dynamic effect (β). For example, when α is null and β = 0.92 (HR of 2.5), Δ = 0.21, whereas when α = 0.92 and β is null, then Δ = 0.42 (see Figure 4).

Table 1 displays the results obtained with the pseudo-*R*^2^s for uncensored cases with various tail defects. These results show that the proposed pseudo-*R*^2^ is the only one able to quantify the dynamic effect. As an example, for a tail defect of 30% with *e*^α^ = 1.25 and *e*^β^ = 2.50, its value is of 37% whereas the highest value for the other indices is 16% (with *OXS*). The same behavior of the proposed pseudo-*R*^2^ is observed for the different values of the tail defect.

**Table 1:** Comparison of the different pseudo-*R*^2^ indices in simulations.

	***e*^α^**	**1**				**1.25**				**1.5**		
**Index**	***e*^β^**	**1**	**2**	**2.5**		**1**	**2**	**2.5**		**1**	**2**	**2.5**
OXS	Mean	0.00	0.03	0.05		0.04	0.14	0.16		0.11	0.25	0.29
	SD	0.01	0.02	0.02		0.02	0.04	0.04		0.03	0.05	0.04
XO	Mean	0.00	0.02	0.03		0.03	0.10	0.12		0.07	0.18	0.21
	SD	0.00	0.01	0.02		0.01	0.03	0.03		0.02	0.03	0.03
N	Mean	0.00	0.02	0.03		0.03	0.10	0.12		0.07	0.18	0.21
	SD	0.00	0.01	0.02		0.01	0.03	0.03		0.02	0.03	0.03
OF	Mean	0.00	0.02	0.03		0.04	0.12	0.14		0.10	0.24	0.26
	SD	0.01	0.02	0.02		0.02	0.04	0.04		0.03	0.04	0.04
RMB	Mean	0.00	0.03	0.04		0.04	0.12	0.14		0.11	0.23	0.25
	SD	0.01	0.02	0.02		0.02	0.03	0.04		0.03	0.04	0.04
Δ	Mean	0.01	0.21	0.27		0.06	0.31	0.37		0.15	0.39	0.45
	SD	0.01	0.04	0.04		0.03	0.04	0.04		0.04	0.04	0.04
**(A) Tail defect of 30%**												
	***e***^**α**^	**1**				**1.25**				**1.5**		
**Index**	***e***^**β**^	**1**	**2**	**2.5**		**1**	**2**	**2.5**		**1**	**2**	**2.5**
OXS	Mean	0.00	0.02	0.02		0.05	0.10	0.11		0.13	0.22	0.24
	SD	0.01	0.01	0.02		0.03	0.04	0.04		0.04	0.05	0.05
XO	Mean	0.00	0.01	0.01		0.02	0.05	0.06		0.07	0.12	0.13
	SD	0.00	0.01	0.01		0.01	0.02	0.02		0.02	0.03	0.03
n	Mean	0.00	0.01	0.01		0.02	0.05	0.06		0.07	0.12	0.13
	SD	0.00	0.01	0.01		0.01	0.02	0.02		0.02	0.03	0.03
OF	Mean	0.00	0.01	0.01		0.05	0.09	0.10		0.13	0.21	0.22
	SD	0.01	0.01	0.01		0.03	0.04	0.04		0.04	0.05	0.05
RMB	Mean	0.00	0.01	0.02		0.05	0.09	0.10		0.14	0.20	0.21
	SD	0.01	0.01	0.02		0.03	0.03	0.04		0.04	0.05	0.05
Δ	Mean	0.01	0.18	0.22		0.06	0.26	0.31		0.15	0.33	0.38
	SD	0.01	0.03	0.03		0.03	0.04	0.04		0.04	0.04	0.04
**(B) Tail defect of 50%**												
	***e***^**α**^	**1**				**1.25**				**1.5**		
**Index**	***e***^**β**^	**1**	**2**	**2.5**		**1**	**2**	**2.5**		**1**	**2**	**2.5**
OXS	Mean	0.01	0.01	0.01		0.05	0.08	0.08		0.14	0.19	0.20
	SD	0.01	0.01	0.01		0.03	0.04	0.04		0.05	0.06	0.06
XO	Mean	0.00	0.00	0.00		0.02	0.02	0.03		0.05	0.07	0.07
	SD	0.00	0.00	0.00		0.01	0.01	0.02		0.02	0.02	0.02
N	Mean	0.00	0.00	0.00		0.02	0.03	0.03		0.05	0.07	0.07
	SD	0.00	0.00	0.00		0.01	0.01	0.02		0.02	0.02	0.02
OF	Mean	0.01	0.01	0.01		0.05	0.07	0.08		0.14	0.18	0.18
	SD	0.01	0.01	0.01		0.03	0.04	0.04		0.05	0.06	0.06
RMB	Mean	0.01	0.01	0.01		0.05	0.07	0.08		0.14	0.17	0.18
	SD	0.01	0.01	0.01		0.03	0.04	0.04		0.05	0.05	0.05
Δ	Mean	0.01	0.17	0.21		0.06	0.24	0.28		0.15	0.31	0.35
	SD	0.01	0.04	0.04		0.03	0.05	0.04		0.05	0.05	0.04
**(C) Tail defect of 70%**.												

*OXS, pseudo-R^**2**^ index from O'Quigley et al. (2005); N, pseudo-R^2^ index from Nagelkerke (1991); XO, pseudo-R^2^ index from Ronghui Xu and O' quigley (1999); OF, pseudo-R^2^ index from O'Quigley and Flandre (1994); RMB, pseudo-R^2^ index from Rouam et al. (2010); **Δ**, the pseudo-R^2^ index we propose in this work; e^α^, propensity effect; e^β^, dynamic effect; SD, standard deviation. Simulations were performed with 500 subjects*.

Table 2 displays the results obtained for the proposed pseudo-*R*^2^ under various tail defects and simulation schemes: (i) Two-part survival model with no censoring and Normally distributed explanatory variables, (ii) Two-part survival model with 20% censoring and Normally distributed explanatory variables, (iii) Two-part survival model with no censoring and Student distributed explanatory variables, (iv) Improper Gompertz model with no censoring and Normally distributed explanatory variables.

**Table 2:** Δ pseudo-*R*^2^ indices in simulations with various simulation designs.

	***e*^α^**	**1**				**1.25**				**1.5**		
**Design**	***e*^β^**	**1**	**2**	**2.5**		**1**	**2**	**2.5**		**1**	**2**	**2.5**
(i)	Mean	0.01	0.21	0.27		0.06	0.31	0.37		0.15	0.39	0.45
	SD	0.01	0.04	0.04		0.03	0.04	0.04		0.04	0.04	0.04
												
(ii)	Mean	0.01	0.22	0.29		0.05	0.31	0.39		0.12	0.40	0.46
	SD	0.01	0.05	0.04		0.03	0.05	0.05		0.04	0.05	0.05
												
(iii)	Mean	0.01	0.21	0.27		0.08	0.32	0.38		0.17	0.41	0.46
	SD	0.01	0.04	0.04		0.03	0.04	0.04		0.04	0.04	0.04
												
(iv)	Mean	0.01	0.19	0.25		0.08	0.31	0.37		0.19	0.41	0.46
	SD	0.01	0.04	0.04		0.03	0.04	0.04		0.04	0.04	0.04
**(A) Tail defect of 30%**												
	***e***^**α**^	**1**				**1.25**				**1.5**		
**Design**	***e***^**β**^	**1**	**2**	**2.5**		**1**	**2**	**2.5**		**1**	**2**	**2.5**
(i)	Mean	0.01	0.18	0.22		0.06	0.26	0.31		0.15	0.33	0.38
	SD	0.01	0.03	0.03		0.03	0.04	0.04		0.04	0.04	0.04
												
(ii)	Mean	0.01	0.18	0.25		0.05	0.27	0.33		0.13	0.35	0.40
	SD	0.01	0.04	0.04		0.03	0.05	0.05		0.04	0.05	0.05
												
(iii)	Mean	0.01	0.18	0.22		0.07	0.27	0.31		0.17	0.35	0.39
	SD	0.01	0.03	0.04		0.03	0.04	0.04		0.04	0.04	0.04
												
(iv)	Mean	0.01	0.17	0.21		0.06	0.26	0.31		0.16	0.34	0.38
	SD	0.01	0.03	0.04		0.03	0.04	0.04		0.05	0.04	0.04
**(B) Tail defect of 50%**												
	***e***^**α**^	**1**				**1.25**				**1.5**		
**Design**	***e***^**β**^	**1**	**2**	**2.5**		**1**	**2**	**2.5**		**1**	**2**	**2.5**
(i)	Mean	0.01	0.17	0.21		0.06	0.24	0.28		0.15	0.31	0.35
	SD	0.01	0.04	0.04		0.03	0.05	0.04		0.05	0.05	0.04
												
(ii)	Mean	0.02	0.17	0.23		0.06	0.24	0.30		0.14	0.33	0.37
	SD	0.02	0.05	0.05		0.04	0.06	0.05		0.06	0.06	0.05
												
(iii)	Mean	0.01	0.17	0.21		0.07	0.25	0.29		0.17	0.32	0.35
	SD	0.01	0.04	0.04		0.04	0.04	0.04		0.05	0.04	0.04
												
(iv)	Mean	0.01	0.17	0.21		0.06	0.24	0.29		0.15	0.31	0.35
	SD	0.01	0.04	0.04		0.04	0.05	0.04		0.05	0.05	0.04
**(C) Tail defect of 70%**												

*e^α^, propensity effect; e^β^, dynamic effect; SD, standard deviation. Simulations were performed with 500 subjects. Detail of the designs displayed in the table : (i) two-part survival model with no censoring and Normally distributed explanatory variables; (ii) Two-part survival model with 20% censoring and Normally distributed explanatory variables (iii) Two-part survival model with no censoring and Student distributed explanatory variables; (iv) Improper Gompertz model with no censoring and Normally distributed explanatory variables*.

In case of 20% censoring, simulation results show that the mean and standard error values of the proposed indice are slightly increased. When looking to the simulation results under an improper Gompertz model, simulation results show that the proposed indice is only slightly affected by model mis-specification. For a Student distribution, we can see that the estimated mean values of the proposed indice are very close to the Normal distribution. Table 3 shows that the estimated mean and standard error values of the proposed indice are slightly increased when the number of subjects decreases. It is worth noting that the slight bias observed for the mean values with censored or reduced samples is linked to the fact that our pseudo-*R*^2^ uses the estimate of the tail defect. In these situations, the dispersion of this nuisance parameter increases.

**Table 3:** Δ pseudo-*R*^2^ indices in simulations with various number of subjects.

	***e*^α^**	**1**				**1.25**				**1.5**		
**Subjects**	***e*^β^**	**1**	**2**	**2.5**		**1**	**2**	**2.5**		**1**	**2**	**2.5**
200	Mean	0.02	0.23	0.29		0.07	0.32	0.38		0.16	0.39	0.45
	SD	0.02	0.06	0.06		0.04	0.07	0.07		0.06	0.07	0.06
500	Mean	0.01	0.21	0.27		0.06	0.31	0.37		0.15	0.39	0.45
	SD	0.01	0.04	0.04		0.03	0.04	0.04		0.04	0.04	0.04
1,000	Mean	0.00	0.20	0.26		0.06	0.30	0.37		0.15	0.39	0.45
	SD	0.00	0.03	0.03		0.02	0.03	0.03		0.03	0.03	0.03
**(A) Tail defect of 30%**												
	***e***^**α**^	**1**				**1.25**				**1.5**		
**Subjects**	***e***^**β**^	**1**	**2**	**2.5**		**1**	**2**	**2.5**		**1**	**2**	**2.5**
200	Mean	0.02	0.20	0.25		0.07	0.27	0.32		0.15	0.33	0.39
	SD	0.02	0.05	0.06		0.05	0.06	0.06		0.06	0.06	0.06
500	Mean	0.01	0.18	0.22		0.06	0.26	0.31		0.15	0.33	0.38
	SD	0.01	0.03	0.03		0.03	0.04	0.04		0.04	0.04	0.04
1,000	Mean	0.00	0.17	0.21		0.05	0.25	0.30		0.15	0.33	0.38
	SD	0.00	0.02	0.03		0.02	0.03	0.03		0.03	0.03	0.03
**(B) Tail defect of 50%**												
	***e***^**α**^	**1**				**1.25**				**1.5**		
**Subjects**	***e***^**β**^	**1**	**2**	**2.5**		**1**	**2**	**2.5**		**1**	**2**	**2.5**
200	Mean	0.04	0.20	0.24		0.08	0.25	0.30		0.16	0.32	0.36
	SD	0.03	0.06	0.06		0.06	0.08	0.07		0.08	0.08	0.07
500	Mean	0.01	0.17	0.21		0.06	0.24	0.28		0.15	0.31	0.35
	SD	0.01	0.04	0.04		0.03	0.05	0.04		0.05	0.05	0.04
1,000	Mean	0.01	0.16	0.20		0.05	0.23	0.28		0.14	0.30	0.34
	SD	0.01	0.03	0.03		0.03	0.03	0.03		0.04	0.04	0.03
**(C) Tail defect of 70%**

*e^α^ : propensity effect; e^β^ : dynamic effect; SD : standard deviation*.

As expected, results obtained for the proposed pseudo-*R*^2^s with and without strata are the same (Table 4).

**Table 4:** Δ pseudo-*R*^2^ index in simulations with strata.

	***e*^α^**	**1**				**1.25**				**1.5**		
**Tail defect**	***e*^β^**	**1**	**2**	**2.5**		**1**	**2**	**2.5**		**1**	**2**	**2.5**
0.3	Mean	0.01	0.22	0.28		0.06	0.31	0.37		0.15	0.39	0.45
	SD	0.01	0.04	0.04		0.03	0.04	0.04		0.04	0.04	0.04
0.5	Mean	0.01	0.19	0.23		0.06	0.26	0.31		0.15	0.33	0.38
	SD	0.01	0.03	0.04		0.03	0.04	0.04		0.04	0.04	0.04
0.7	Mean	0.01	0.18	0.23		0.06	0.25	0.29		0.15	0.30	0.35
	SD	0.01	0.04	0.04		0.04	0.05	0.05		0.05	0.05	0.04

*e^α^, propensity effect; e^β^, dynamic effect; SD, standard deviation*.

It should be noted that comparison of the values of the pseudo-*R*^2^ across different tail defect values are difficult to interpret and can be misleading since the dynamic/propensity effects should be interpreted conditionally upon the defective survival distributions that are not normalized to one but to different values according to the defect. This is also the case for the other indices that increase when the proportion of susceptible subjects increase. For example, with *e*^α^ = 1.5 and *e*^β^ = 2.5, values of the *OXS* are 29, 25, and 20% for a tail defect of 30, 50, and 70%, respectively.

### 3.2. CARTaGENE Results

Among the 4,554 women selected for the analysis, 60 (1.32%) had breast cancer during the 5 years of follow-up. The PRS' mean (Evan's score) was higher among participants with a diagnosed breast cancer within the 5 years (0.57) than for those free of event at 5 years (0.44).

**Figure 5:**
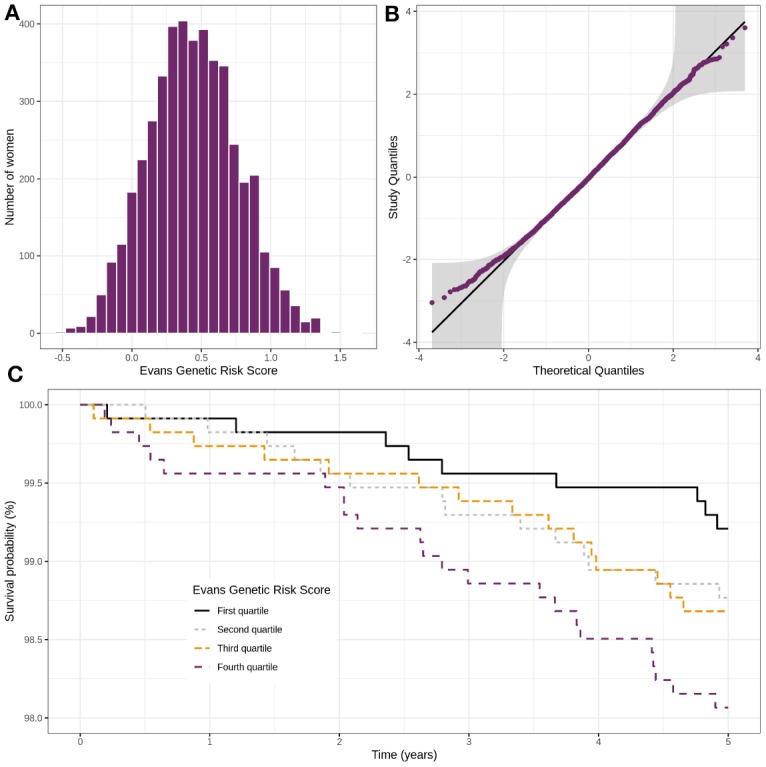
Distribution of Evans' polygenic risk score in the CARTaGENE cohort (*n* = 4,554) **(A)**, control women QQ-plot with confidence bands based on an inversion of the Kolmogorov–Smirnov test **(B)** and incidence of breast cancer in the cohort according to the Evans' score **(C)**.

Figure 5 displays the distribution of the PRS in the cohort, the normal quantile-quantile plot and the Kaplan-Meier estimate of the probability of not experiencing breast cancer for the four groups based on the PRS quartiles. A seen from Figures 5A,B, the distribution of the PRS can be considered close to the Normal distribution. From Figure 5C, we can see that higher the PRS, higher the risk of breast cancer is.

Table 5 shows the results for Δ and the five pseudo-*R*^2^ indices. For these latter, pseudo-*R*^2^ measures are computed upon an entry-age-stratified age-scaled Cox survival model. Δ was the highest pseudo-R^2^ (17.8%), while *OF*, *RMB*, and *OXS* were equal to 12.0, 12.1, and 14.0%, respectively. The indices *XO* and *N* had values of 0.21 and 1.01%, respectively.

**Table 5:** Pseudo-*R*^2^ index values for Evans genetic risk score in the CARTaGENE cohort (*n* = 4,554).

	**Pseudo-*R*^**2**^ (%)**
OXS	14.1
N	1.01
XO	0.21
OF	12.0
RMB	12.1
Δ	17.8

*OXS, pseudo-R^2^ index from O'Quigley et al. (2005); N, pseudo-R^2^ index from Nagelkerke (1991); XO, pseudo-R^2^ index from Ronghui Xu and O' quigley (1999); OF, pseudo-R^2^ index from O'Quigley and Flandre (1994); RMB, pseudo-R^2^ index from Rouam et al. (2010); **Δ**, the pseudo-R^2^ index we propose in this work*.

## Discussion

The evaluation of the PRS for clinical prediction has received a lot of attention these last years. For binary or time-to-event traits, prediction accuracy of PRS is usually assessed using likelihood-based pseudo-*R*^2^ criteria that can be complicated to compute for complex models. In this work, we have proposed a novel pseudo-*R*^2^ test for assessing the accuracy prediction over a time period of PRS for both the probability of occurrence of the event of interest and the dynamic. This criterion is easy to compute since it avoids maximizing Cox's partial likelihood under the alternative.

As seen from the simulation study, our pseudo-*R*^2^ showed good performances as compared to classical indices based on the Cox proportional hazards model. As expected, it showed very good performances when there is a dynamic effect related to the PRS. Moreover, when the main effect of the PRS is on the dynamic of the event among the susceptible subjects, the proposed pseudo-*R*^2^ is the only one able to quantify this effect. Our simulations have shown the good behaviors of the indices proposed by O'Quigley and Flandre (based on Schoenfeld residuals) (O'Quigley and Flandre, 1994), by O'Quigley et al. (based on explained randomness measures) (O'Quigley et al., 2005), and by Rouam et al. (2010). In contrast, the coefficient proposed by Nagelkerke (1991) that is frequently used in the literature showed poor results. Simulations results have shown that the proposed indice is only barely affected by model misspecification and covariate distribution. It slightly increases with reduced sample size or censoring. Based on our simulation study, when a dynamical effect related to the PRS is expected, we recommend using our proposed pseudo-*R*^2^. In other cases, our index can be used together with the *OF* and *OXS* indices since they show good performances. In contrast, we do not recommend the use of the Nagelkerke index for quantifying predictive accuracy of PRS for time to event outcomes.

Notwithstanding the good performance of the proposed criteria, it has some shortcomings that should be mentioned. We should first keep in mind that pseudo-*R*^2^s are always model-based criteria. In our case, our pseudo-*R*^2^ measures the proportion of variability in the outcome that is explained by the PRS under the assumption that the PRS may be linked to the dynamic and/or the propensity of the occurrence of the event. In this work, it relies on a two-component survival model which is potentially prone to misspecification. Thus, such underlying assumption of a mixture model should be discussed before considering the use of the proposed pseudo-*R*^2^. We do not claim that our model represents the reality but that it is a useful approximation of what we suppose the effects (propensity/dynamic) are. Moreover, it is also worth noting that pseudo-*R*^2^s and even the classical *R*^2^ are not robust to outlier observations and non-normal distributions. Thus, we should warn practitioners to be cautious and examine the PRS distribution. Here, we have considered pseudo-*R*^2^ criteria since our main interest was to focus on the percentage of variation in the outcome explained by the PRS. Time-dependent ROC curves (with incident cases/dynamic controls; Heagerty and Zheng, 2005) could also have been considered if the main objective was to evaluate the prognostic potential of the PRS by focusing on the correct classification rates. Moreover, it should be noted that our pseudo-*R*^2^ is not designed for more complex situations such as those with marker-dependent censoring. We plan to do further work in this direction.

This novel pseudo-*R*^2^ was used to evaluate the 5-years predictive accuracy of the PRS proposed by Evans et al. (2017) for breast cancer occurrence on the CARTaGENE cohort. In this study, we reported a pseudo-*R*^2^ of 17.8% for our novel index which is higher than the values reported by *OF* and *OXS* indices. Based on the results from our simulation study, we hypothesize that the PRS has both a propensity effect and a dynamical effect. This is not surprising since the selected SNP are located within genes that encode for proteins that are involved in important processes such as cell growth and division. It is worth noting that for this analysis we have considered an age-dependent model with four strata. Here, our pseudo-*R*^2^ provides a global predictive measure that average all differences for both the propensity and dynamic effects taking into account the stratum of age at entry. Other strategies can be considered and easily implemented. We should keep in mind that the majority of PRS were developed and evaluated from case-control designs which raises some issues about misspecification when applying these results for time-to-event prediction (Lambert et al., 2019). With large prospective cohorts, we may expect to see in a close future more published polygenic hazard scores.

Finally, we think that the proposed novel pseudo-*R*^2^, which is easy to implement with standard softwares, is worth being used to evaluate PRS for predicting incident events in cohort studies.

## Data Availability Statement

The data analyzed in this study were collected in the context of the CARTaGENE cohort. The data are available upon official request and ethical approval. The dataset cannot be made publicly available but can be obtained by interested researchers upon request to the CARTaGENE team at CHU Sainte-Justine. The requests can be made using the CARTaGENE online application at sdas.cartagene.qc.ca, by contacting the CARTaGENE team at access@cartagene.qc.ca or by calling 514-345-2156.

## Ethics Statement

CARTaGENE has obtained ethics approval by the CHU Sainte-Justine under the reference: MP-21-2011-345, 3297. The latest annual ethics renewal was granted on September 13, 2019.

## Author Contributions

JD and PB developed the original statistic. PB coordinated the project and is JD's Ph.D. thesis advisor. JD, RJ, and PB analyzed the data. TD, YP, CL, and NN participated in the collection of the data. JD, RJ, TD, YP, CL, NN, and PB participated in writing the original draft. All authors read and approved the final manuscript.

## Conflict of Interest

The authors declare that the research was conducted in the absence of any commercial or financial relationships that could be construed as a potential conflict of interest.
