# Supplementary material for: Quantifying the Predictive Accuracy of a Polygenic Risk Score for Predicting Incident Cancer Cases : Application to the CARTaGENE Cohort
Source: Front Genet. 2020 Apr 24;11:408. doi: 10.3389/fgene.2020.00408 (PMC7193029; doi:10.3389/fgene.2020.00408)
Supplement: Supplementary file 1 [file Data_Sheet_1.pdf]

Supplemental Data

We denote $\hat{\rho}^2\left(W_1^*, W_2^*\right) = \hat{\rho}^2$ the square of the correlation coefficient between W_1^* and W_2^* that takes values between 0 and 1.

$$0 \leq \hat{\rho}^2 = \frac{\hat{\sigma}(W_{12}^*)^2}{\hat{\sigma}^2(W_1^*)\hat{\sigma}^2(W_2^*)} \frac{(\sum W_{1i}^* W_{2i}^* - \sum W_{1i}^* \sum W_{2i}^*/k)^2}{\left(\sum {W_{1i}^*}^2 - (\sum W_{1i}^*)^2/k\right)\left(\sum {W_{2i}^*}^2 - (\sum W_{2i}^*)^2/k\right)} \leq 1$$

Then, we have:

$$0 \leq \frac{\sum(W_{1i}^* W_{2i}^*)^2 - \frac{2\sum W_{1i}^* W_{2i}^* \sum W_{1i}^* \sum W_{2i}^*}{k} + \frac{(\sum W_{1i}^*)^2(\sum W_{2i}^*)^2}{k^2}}{\sum {W_{1i}^*}^2 \sum {W_{2i}^*}^2 - \frac{\sum {W_{1i}^*}^2(\sum W_{2i}^*)^2}{k} - \frac{\sum {W_{2i}^*}^2(\sum W_{1i}^*)^2}{k} + \frac{(\sum W_{1i}^*)^2(\sum W_{2i}^*)^2}{k^2}} \leq 1$$

Thus:

$$0 \leq \frac{\frac{\sum {W_{1i}^*}^2(\sum W_{2i}^*)^2}{k} + \frac{\sum {W_{2i}^*}^2(\sum W_{1i}^*)^2}{k} - \frac{2\sum W_{1i}^* W_{2i}^* \sum W_{1i}^* \sum W_{2i}^*}{k}}{\sum {W_{1i}^*}^2 \sum {W_{2i}^*}^2 - \sum(W_{1i}^* W_{2i}^*)^2} \leq 1$$

$$0 \leq \frac{\sum {W_{1i}^*}^2(\sum W_{2i}^*)^2}{k} + \frac{\sum {W_{2i}^*}^2(\sum W_{1i}^*)^2}{k} - \frac{2\sum W_{1i}^* W_{2i}^* \sum W_{1i}^* \sum W_{2i}^*}{k} \leq \sum {W_{1i}^*}^2 \sum {W_{2i}^*}^2 - \sum(W_{1i}^* W_{2i}^*)^2$$

Finally after rearranging, we obtain that:

$$0 \leq \frac{\det(\Sigma) - \det(\Sigma^\star)}{\det(\Sigma)} \leq 1.$$
